# The Most Popular Book Man in Texas

**Published:** 1911-02

**Authors:** 


Constipation is a prolific source of disease. Neglect of the most important function,—evacuation—often results in atony of the intestines. The Lapactic pills of Sharp & Dohme is the only cure for constipation that I know. They contain strychnine, aloes and podophyllin, and do not purge or gripe nor leave the bowels torpid. They are indeed a luxury.

---

Abbott, the Hard Hitter, has excelled all former efforts in his January issue of the *American Journal of Clinical Medicine.* It's a wonder. Ask for it and subscribe at once. With such a staff as Drs. A. S. Burdick, W. F. Waugh, Achard, Canler, Clough et al., it is not surprising, for each one is a trained journalist, a hard worker and a royal good fellow.

---

The "Revised" State Board of Health of Texas is composed of Drs. Ralph Steiner, of Austin, President; B. F. Calhoun, of Beaumont; B. M. Worsham, of El Paso; A. W. Fly, of Galveston; S. M. Lister, of Houston (vice Herff, declined); K. H. Beall, of Fort Worth, and H. L. McLaurin, of Dallas.

---

The Most Popular Book Man in Texas.—Geo. Henser, of Corsicana, who for many years has represented the Appletons in Texas, has left their service (there is where the Appletons "dropped their money purse"), and formed a copartnership with Dr. J. A. Majors—firm J. A. Majors & Co., New Orleans. They will represent W. B. Saunders Co., of Philadelphia, and will keep on hand a large stock of medical books. I hope Dr. Henser will not cease his pleasant visits to Texas. We shall miss him. W. B. Saunders Co. are getting out a book on Surgery by the famous Mayo brothers. Dr. Henser will have the handling of it. Write him.

---

THE MEDICAL COUNCIL of Philadelphia, Dr. J. J. Taylor, editor, in its "Business Department" each month gives the doctors some most valuable pointers. *The Council* is a living entity. Send for a sample copy.

---

"EVERY DOCTOR'S ERGOT" again makes its bow to "the Red Back Family." Same old Ergotole. Just as good as it ever was, and it always has been the *King* of Ergots. With an ounce bottle of it in your obstetric bag and a good clean hypodermic syringe with a clean sharp needle you are armed for any hemorrhage. Use it hypodermically when you want quick results. Use it by the mouth when that method is preferable. For internal use it is always acceptable to your patient because of its pleasant taste. It never nauseates the patient. In that respect is is the extreme opposite of the fluid extract.. For that matter, it is better in every other respect. We take it that all of our older readers use Ergotole. To some of our younger readers it may not be so well known, and to these friends we suggest sending for a sample. Tell them that Daniel recommended it to you.

